# Low Molecular Weight Fucoidan against Renal Ischemia–Reperfusion Injury via Inhibition of the MAPK Signaling Pathway

**DOI:** 10.1371/journal.pone.0056224

**Published:** 2013-02-13

**Authors:** Jihui Chen, Weiling Wang, Quanbin Zhang, Fei Li, Tianluo Lei, Dali Luo, Hong Zhou, Baoxue Yang

**Affiliations:** 1 Department of Pharmacology, School of Basic Medical Sciences, Peking University, and Key Laboratory of Molecular Cardiovascular Sciences, Ministry of Education, Beijing, P.R. China; 2 Institute of Oceanology, Chinese Academy of Sciences, Qingdao, P.R. China; 3 Department of Pharmacology, Capital Medical University, Beijing, P.R. China; Universidade de Sao Paulo, Brazil

## Abstract

**Background:**

Ischemia reperfusion injury (IRI) is a leading cause of acute kidney injury (AKI) in both native and transplanted kidneys. The objective of the present study was to evaluate whether low-molecular-weight fucoidan (LMWF) could attenuate renal IRI in an animal model and *in vitro* cell models and study the mechanisms in which LMWF protected from IRI.

**Methodology/Principal Findings:**

Male mice were subjected to right renal ischemia for 30 min and reperfusion for 24 h, or to a sham operation with left kidney removed. Kidneys undergone IR showed characteristic morphological changes, such as tubular dilatation, and brush border loss. However, LMWF significantly corrected the renal dysfunction and the abnormal levels of MPO, MDA and SOD induced by IR. LMWF also inhibited the activation of MAPK pathways, which consequently resulted in a significant decrease in the release of cytochrome c from mitochondria, ratios of Bax/Bcl-2 and cleaved caspase-3/caspase-3, and phosphorylation of p53. LMWF alleviated hypoxia-reoxygenation or CoCl_2_ induced cell viability loss and ΔΨm dissipation in HK2 renal tubular epithelial cells, which indicates LMWF may result in an inhibition of the apoptosis pathway through reducing activity of MAPK pathways in a dose-dependent manner.

**Conclusions/Significance:**

Our *in vivo* and *in vitro* studies show that LMWF ameliorates acute renal IRI via inhibiting MAPK signaling pathways. The data provide evidence that LMWF may serve as a potential therapeutic agent for acute renal IRI.

## Introduction

Ischemia reperfusion injury (IRI) is a leading cause of acute kidney injury (AKI) in both native and transplanted kidneys. As a common pathophysilological cause of acute renal failure (ARF), IRI-induced AKI occurs in many clinical settings including renal transplantation, shock, and vascular surgery [1]. However, no effective therapy for AKI beyond supportive treatment is currently available [2]. Accumulated evidences indicate that apoptotic pathways are involved in IRI and AKI [3]. Clinical and experimental studies have shown that the tissue damage and cell apoptosis that occur following IR, especially during reperfusion, are due in part to oxidative stress injury [4].

The oxidative stress injury caused by IRI has been attributed to the activation of mitogen activated protein kinase (MAPK) pathways including extracellular signal-regulated kinase (ERK), c-Jun N-terminal kinase (JNK) and p38. ERK promotes cell survival while JNK and p38 lead to cell death. p38 MAPK serves as a nexus for signal transduction and plays a pivotal role in numerous biological processes. Activation of JNK/p38, via the activation of apoptosis signal-regulating kinase 1 (ASK1), a MAP kinase kinase kinase, plays a key role in cytokine- and stress-induced apoptosis [5]. Some studies postulate that the balance between ERK versus JNK/p38 may determine the fate of the cell [6], [7].

Fucoidan represents an intriguing group of natural fucose-enriched sulfated polysaccharides. Marine brown algae are one of the richest sources of sulfated polysaccharides. The brown seaweed Laminaria japonica is common seafood in China, Japan, and South Korea. It has also been a traditional Chinese medicine for more than 1000 years. Fucoidan is available as food supplement in Japan and United States. A fraction of low-molecular-weight fucoidan (LMWF) (∼7 kDa) is obtained by radical depolymerization of extracts from brown seaweed. The use of LMWF is presently expected to be safe up to 2000 mg/kg body weight per day in rats [8], [9], and presents no significant genotoxic concern [10].

LMWF exhibits various biological activities including anti-viral, anti-angiogenic, anti-tumoral, anti-thrombotic, anti-coagulant, antiadhesive, and anti-inflammatory effects [11], [12], [13], [14]. In the literature, LMWF inhibited smooth muscle cell proliferation and intimal hyperplasia and prevented in-stent restenosis and transplanted arteriosclerosis [15]. Fucoidan treatment promoted neovascularization and angiogenesis in rat models of critical hind limb ischemia [16]. Moreover, the protective effect of fucoidan in myocardial IRI has been described previously [17], [18]. Fucoidan also had renoprotective effects in chronic renal failure model in rats [19], [20]. Therefore, we proposed that LMWF might be a possible candidate for treating acute renal IRI.

In present study, we studied whether LMWF exerted a protective role against acute renal IRI using an *in vivo* mouse model and *in vitro* cell models, as well as its possible protective mechanisms in both situations. The experimental results showed LMWF prevented the IRI in a dose-dependent manner, which indicates that LMWF may be developed into a candidate drug for preventing or treating renal IRI.

## Materials and Methods

### Source of LMWF

LMWF was isolated from L. japonica commercially cultured in Qingdao, China, as described previously [19]. Its average molecular weight is about 7000 determined by high performance steric exclusion chromatography analysis. LMWF was dissolved in physiological saline for animal treatment and in PBS for cell incubation.

### Ethics statement

This study was carried out in strict accordance with the recommendations in the Guide for the Care and Use of Laboratory Animals of China Association for Laboratory Animal Science. All animal care and protocols were approved by the Animal Care Committee of Peking University Health Science Center. All sacrifice was performed under pentobarbitone anesthesia, and every effort was made to minimize animal suffering.

### Animal models of acute renal injury

Male C57BL/6J mice (8–10 weeks old), weighting 20–22 g, were purchased from the Animal Center of Peking University Health Science Center. The animals were housed with a 12/12 h light/dark cycle and food and water available ad libitum. The mice were randomly divided into four groups: sham-operated group; sham-operated with LMWF-treated group; IR group; IR with LMWF-treated group. For the warm renal IRI, the mice were anesthetized by intraperitoneal injections of sodium pentobarbital (80 mg/kg). Then, the renal pedicle was exposed by flank incision. Right renal pedicle was clamped for 30 min, and the left kidney was removed. For reperfusion, the clamp was released and the kidney was monitored for color change to confirm blood reflow before suturing the incision. For sham control, animals were operated similarly without renal pedicle clamping. In LMWF-treated groups, 100 mg/kg/day of LMWF was intraperitoneally injected from 7 days before renal IR until sacrifice. Mice in sham-operated group and IR group received physiological saline as control.

### Hematoxylin-eosin staining and TUNEL assay

Kidneys after reperfusion for 24 h were harvested and fixed with 4% formaldehyde for paraffin embedding. Paraffin-embedded tissues were sectioned at 7 µm for hematoxylin and eosin staining. Histological changes were evaluated by analyzing the percentage of renal tubules that displayed cell lysis and brush border loss. The development of tissue damage was scored by double blinding as follows: 0, no damage; 1, <25%; 2, 25–50%; 3, 50–75%; 4, >75%. Representative fields were pictured.

TUNEL assay was also conducted using the *in situ* Cell Death Detection kit (Roche Applied Science) following the manufacturer's instruction. Positive staining in cell nucleus with DNA breakage was identified under fluorescence microscopy. The apoptotic index was defined as (number of apoptotic cells/total number of nucleated cells) ×100.

### Measurement of blood creatinine, urea nitrogen and LDH

Renal function was monitored by measuring blood creatinine and urea nitrogen (BUN). After reperfusion for 24 h, blood samples were collected for determination of urea, creatinine, and LDH. Blood creatinine and LDH concentrations were measured with commercial kits (NJJC Bio), according to the manufacture's instructions. BUN concentration was measured using quantitative colorimetric urea determination kit (QuantiChrom urea assay kit-DIUR-500).

### Myeloperoxidase (MPO), SOD and MDA assays

The kidney tissue MPO, SOD and MDA levels were measured using commercial kits (NJJC Bio), according to the manufacture's instructions.

### Cell culture

HK2 cells (human kidney proximal tubular cells) were purchased from Cell Culture Centre, Institute of Basic Medical Science Chinese Academy of Medical Sciences (Beijing, China). Briefly, HK2 cells were cultured in DMEM/F12 containing 10% fetal bovine serum (FBS; Hyclone), 2 mM glutamine, 100 U/ml penicillin and 100 µg/ml streptomycin, in a humidified atmosphere with 5% CO_2_ at 37°C.

For hypoxia-reoxygenation protocol, hypoxia was achieved by using the MGC AnaeroPack System (Mitsubishi Gas Chemical Co., Tokyo, Japan), which was equipped with an AnaeroPack, disposable O_2_-absorbing and CO_2_-generating agent, and an indicator to monitor oxygen depletion. The AnaeroPack jar is capable of depleting the concentration of O_2_ down to <0.1% in 30 min and of providing a 21% CO_2_ atmosphere. Cells were cultured to 70% to 80% confluence and then they were serum deprived for 24 h, then cells were cultured for 12 h in HBSS without glucose, in a hypoxic atmosphere. After hypoxia, cells were maintained in complete medium with 1∼20 µg/ml LMWF (diluted from a 20 mg/ml stock solution in PBS) or MAPK inhibitors, U-0126 (Calbiochem), SB-203580 (Calbiochem), SP-600125 (Biomol), at 15 µM (diluted from a 15 mM stock solution in DMSO) and 21% O_2_ and 5% CO_2_ for reoxygenation for 3 h. The control cells were incubated at 37°C in an atmosphere of 21% O_2_ and 5% CO_2_ for the same duration as hypoxic cells.

For chemical hypoxia protocol, cells were cultured to 70% to 80% confluence, and then pretreated with LMWF or MAPK inhibitors for 1 h, and then cells were cultured in serum-free DMEM/F12 with 600 µM cobalt chloride (CoCl_2_). The control cells were incubated without LMWF and CoCl_2_.

### Cell viability assay

Cell viability assay was performed according to the manufacturer's instruction of CCK-8 kit (Dojindo). HK2 cells were plated in 96-well plates at a density of 5000 cells/well. When cells were grown to 70% to 80% confluence, chemical hypoxia protocol or hypoxia-reoxygenation protocol was performed. Then CCK-8 solution at a dilution of 1/10 with 10% FBS DMEM/F12 was added to each well and the cells were incubated for 3 h at 37°C. Absorbance at 450 nm was measured with a microplate reader (Biotek, MQX200).
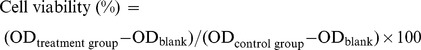



### Annexin V-FITC/PI assay

Cell apoptosis was analyzed by staining with annexin V fluorescein isothiocyanate (annexin V-FITC) and propidium iodide (PI) staining. HK2 cells were collected, washed in PBS and resuspended in 200 µl of binding buffer containing 10 µl of annexin V-FITC and incubated for 15 min at room temperature in the dark. 5 µl of PI and 300 µl of binding buffer were added to each sample before flow cytometric analysis. Cells were analyzed using a FACS calibur (Becton Dickinson). In each sample, a minimum of 10,000 cells were counted. Data analysis was performed using Cell Quest software (Becton Dickinson).

### Measurement of mitochondrial membrane potential

The fluorescent, lipophilic and cationic probe, JC-1 (Beyotime), was employed to measure the mitochondrial membrane potential (Δψm) of HK2 cells according to the manufacture's instructions. HK2 cells were plated in 96-well plates at a density of 5000 cells/well. When cells were grown to 70% to 80% confluence, chemical hypoxia protocol or hypoxia-reoxygenation protocol was performed. Then cells were incubated with JC-1 staining solution for 20 min at 37°C. The fluorescence was detected with a Fluostar Optima microplate reader (BMG Technologies). The wavelengths of excitation and emission were 490 nm and 535 nm for detection of monomeric form of JC-1. 525 nm and 590 nm were used to detect aggregation of JC-1. The ratio of ‘red’ to ‘green’ fluorescence represented ΔΨm of HK2 cells.

### Western blot analysis

Tissues or cells were homogenized in RIPA lysis buffer containing protease inhibitor cocktail (Roche). Mitochondrial and cytosolic proteins were isolated using the Mitochondria/Cytosol Fractionation Kit according to the manufacturer's protocol (Beyotime Inst Biotech). Total protein was measured by BCA (Pierce) and size separated by sodium dodecyl sulfate–polyacrylamide gel electrophoresis. Proteins were blotted to polyvinylidene difluoride membranes (Amersham Biosciences). Blots were incubated with antibodies against Bax, Bcl-2, p-ERK, ERK2, p-JNK, JNK2, c-fos and β-actin (Santa Cruz), p-p38, p38, p-CREB, CREB and caspase-3 (Cell Signaling Technology), p-p53 (ser 392), cytochrome-c (Epitomics), p53 (Sigma), HIF-1α (Abcam). Goat anti-rabbit IgG and goat anti-mouse IgG (Santa Cruz) were added and the blots were developed with ECL plus kit (Amersham Biosciences).

### Statistical analyses

All results are expressed as mean ± SEM. For multiple comparisons, the statistical analysis was performed by using one-way ANOVA followed by the Tukey's multiple comparison tests. *P*-values<0.05 were considered statistically significant.

## Results

### LMWF protected kidney from IRI

**Figure 1:**
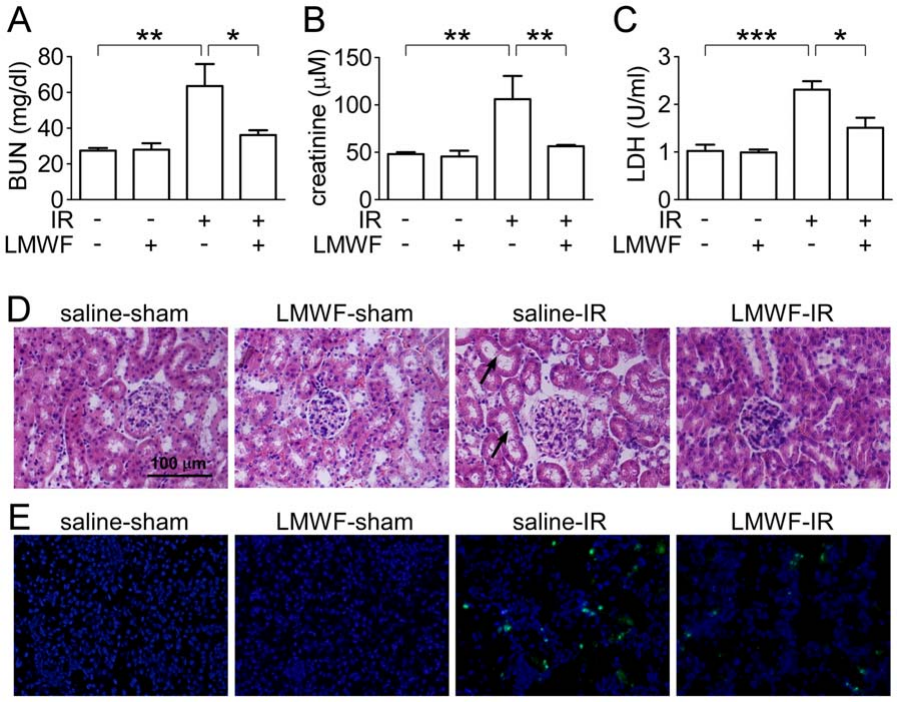
LMWF reduced renal functional defect and tissue damage caused by IR. C57BL/6J mice were administered with vehicle or LMWF daily for 7 days before surgery. Serum and kidneys were collected for renal function test and histological examination after reperfusion for 24 h. (A) Serum BUN. (B) Serum creatinine concentration. (C) Serum LDH activity. (D) Representative images of H&E staining of kidney (original magnification ×400). Arrow: lysed tubules. (E) Representative images of TUNEL (green fluorescence) and Hochoest (Nuclei) staining of kidney (original magnification ×400). Data are presented as mean ± SEM. n = 5–8 for each group. *P<0.05; **P<0.01; ***P<0.001.

To clarify the role of LMWF in renal IRI, C57BL/6j mice were subjected to sham operation, or warm ischemia (renal artery occlusion for 30 minutes) followed by 24 h reperfusion with treatment of saline or 100 mg/kg/day LMWF. IR induced AKI showing the renal dysfunction with significant increases in blood creatinine and BUN. LMWF significantly prevented renal IRI, as demonstrated by reduced blood creatinine and BUN compared with IR group (Fig. 1A and 1B). LMWF markedly reduced high blood LDH level after reperfusion for 24 h (Fig. 1C). LMWF did not increase BUN, creatinine and LDH levels in sham-operated group. We have also tested a group of mice (n = 8) receiving only 100 mg/kg of LMWF after suturing the incision, blood creatinine and urea nitrogen were measured after reperfusion for 24 h. The mice treated with LMWF post-ischemia showed lower blood creatinine (81.5±13.0 µM) and BUN (48.7±4.3 mg/dl), but not significantly different compared with IR group: creatinine (108.2±17.1 µM) and BUN (65.0±11.8 mg/dl).

Renal tissue morphology is shown in Fig. 1D. In sham-operated control and sham-operated with LMWF-treated groups, renal tissues were normal. The kidneys in IR group showed the acute tubular damage in proximal tubules, including tubular dilatation and brush border loss (arrow). LMWF treatment protected the tubular epithelium from swelling and brush border loss. The pathological scores were significantly different between the renal IRI model (2.4±0.2) and LMWF-treated group (1.2±0.4). As expected, no tubular injury was found in two sham groups.

**Figure 2:**
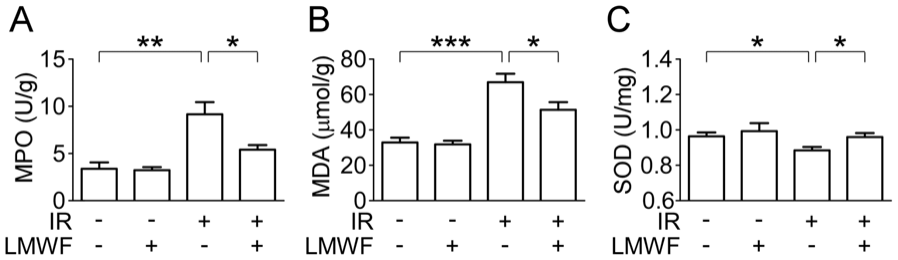
LMWF prevented renal oxidative stress and lipid peroxidation after IR. Kidneys after reperfusion for 24 h were collected for measurement. (A) MPO activity in renal tissue. (B) MDA concentration in renal tissue. (C) SOD activity in renal tissue. Data are presented as mean ± SEM. n = 5–8 for each group. *P<0.05, **P<0.01, ***P<0.001.

Renal IRI significantly increased MPO activity, MDA content and decreased SOD activity (Fig. 2A, 2B and 2C). MPO activity, which is accepted as an indicator of neutrophil infiltration, was significantly higher in the kidney tissue of the saline-treated IR group than that of the sham group. On the other hand, LMWF treatment significantly decreased renal tissue MPO level (Fig. 2A). The oxidative abnormalities were obviously ameliorated by the LMWF treatment, which manifested as the significant reduction of MDA content and the increase of SOD activity (Fig. 2B and 2C). There was no significant difference in tissue MPO, SOD and MDA levels between saline- and LMWF-treated mice in the sham-operated groups.

### LMWF inhibited IR induced renal cell apoptosis

**Figure 3:**
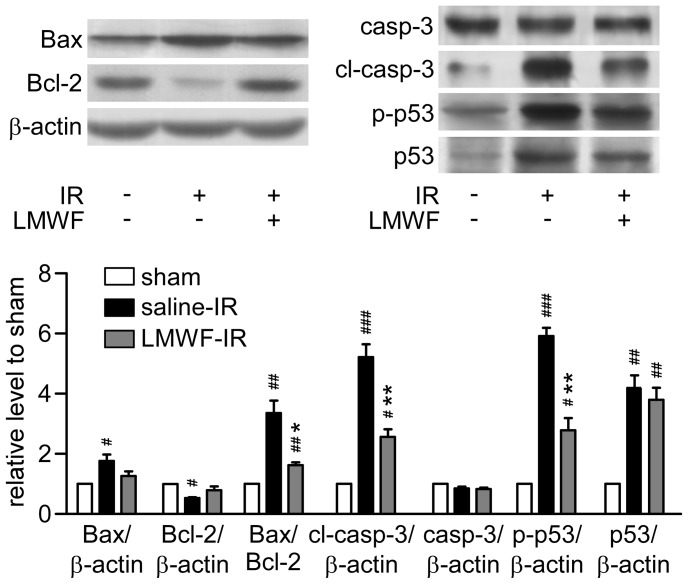
LMWF reduced cell apoptosis in kidney after IR. Protein expression levels of kidneys after reperfusion for 24 h were detected by Western blot analysis. Representative blotting (*up*) and quantification of protein levels (*down*) are shown. Mean ± SEM. n = 3; #*P*<0.05, ##*P*<0.01, ###*P*<0.001 vs. sham, *P<0.05, **P<0.01 vs. saline treated IR group.

The apoptotic cells in the kidney were observed with TUNEL assay. TUNEL positive cells (apoptotic index: 8.9±1.8) in mice subjected to ischemia followed by 24 h reperfusion were significantly more than those of sham mice (apoptotic index: 0.6±0.1). However, fewer TUNEL positive cells were visible in mice treated with LMWF (apoptotic index: 4.5±0.4, Fig. 1D). The data suggested that LMWF protected the kidney from renal tubular apoptosis. As shown in Fig. 3, after reperfusion for 24 h, the expression of pro-apoptotic protein Bax was increased, whereas the expression of anti-apoptotic protein Bcl-2 was decreased. It was also found that the cleaved caspase-3 was decreased and procaspase-3 increased. The relative expression ratios of these proteins are shown in Fig. 3. LMWF significantly reduced ratios of Bax/Bcl-2, cleaved caspase-3/procaspase-3 compared to the IR group. We also found that p53 activation was involved in IR-induced cell apoptosis, as evidenced by increase of p-p53 (ser 392) and p53 levels. Notably, pretreatment with LMWF remarkably inhibited the phosphorylation of p53, but did not affect the expression of p53.

### LMWF inhibited IR activated MAPK pathways

**Figure 4:**
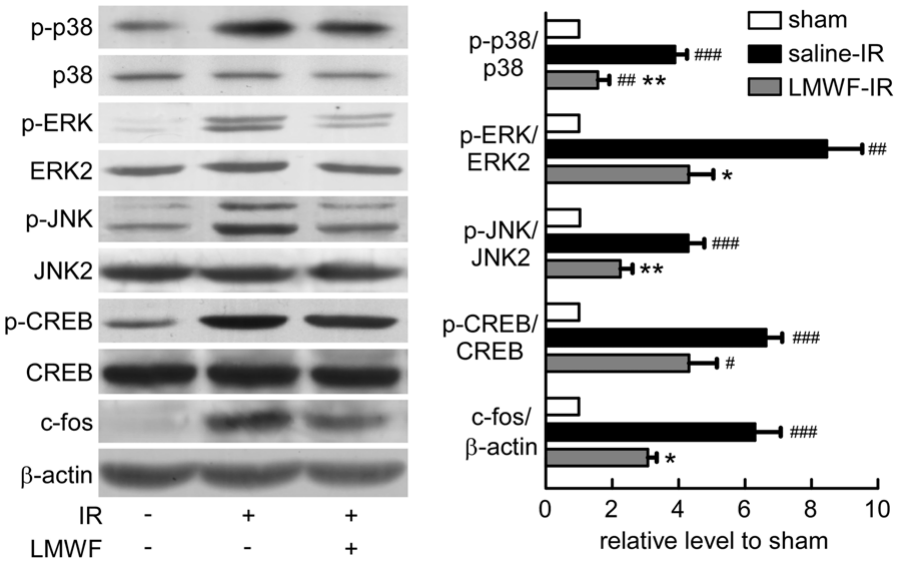
LMWF inhibited MAPK signaling pathway in kidney after IR. Expression levels of ERK, p38, JNK, CREB and c-fos of kidneys after reperfusion for 2 h were detected by Western blot analysis. Representative blotting (*left*) and quantification of protein levels (*right*) are shown. Mean ± SEM. n = 3; #*P*<0.05, ##*P*<0.01, ###*P*<0.001 vs. sham, *P<0.05, **P<0.01 vs. saline treated IR group.

The effects of IR on the phosphorylation of JNK, p38, ERK1/2, CREB and the expression of c-fos were detected. Kidneys after reperfusion for 2 h were collected and used for Western analysis. As shown in Fig. 4, IR promoted the phosphorylation of JNK, p38, ERK1/2 and CREB, and increased c-fos expression after reperfusion for 2 h. LMWF significantly inhibited IRI-induced activation of MAPK pathways and CREB and reduced c-fos expression.

### LMWF protected HK2 cell from apoptosis induced by hypoxia-reoxygenation

**Figure 5:**
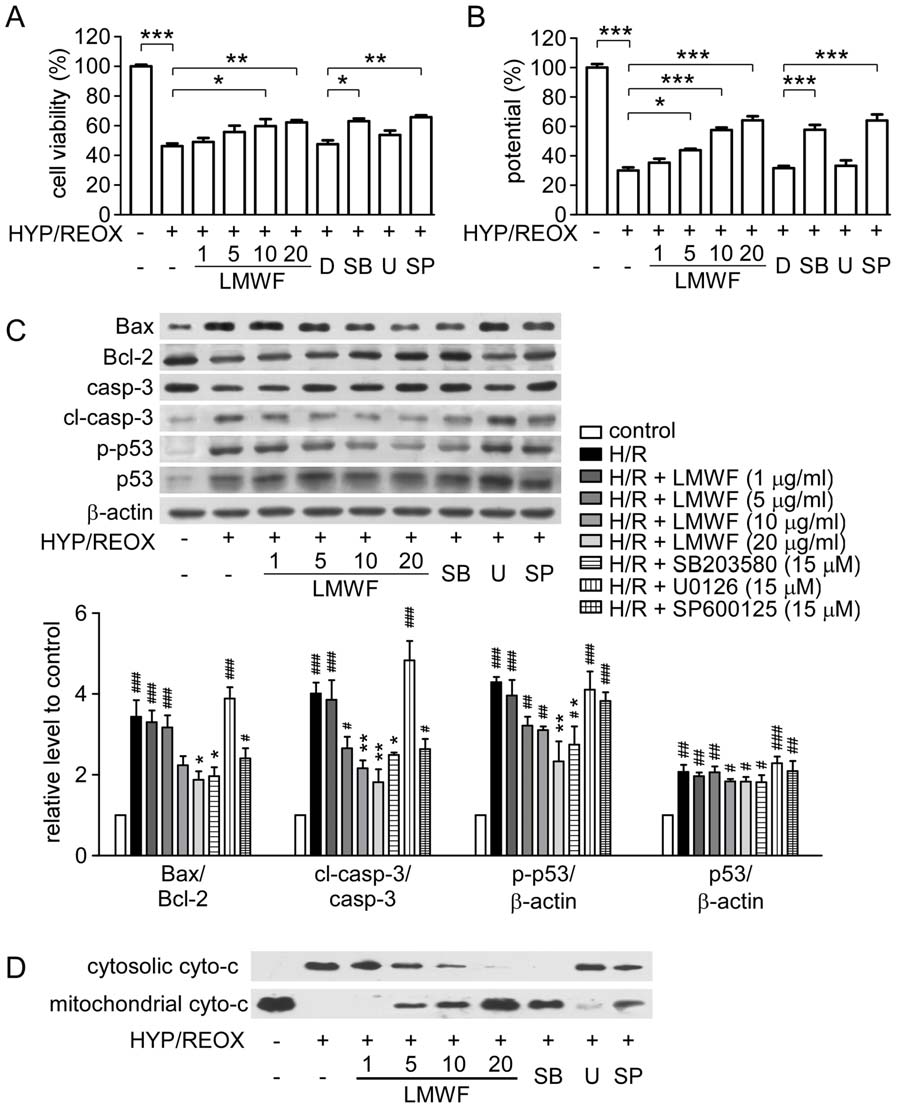
LMWF protected HK2 cells during hypoxia-reoxygenation. HK2 cells were exposed to hypoxia for 12 h by using the MGC AnaeroPack System. After hypoxia, cells were maintained in complete medium for 3 h with 1∼20 µg/ml LMWF or MAPK inhibitors, SB-203580 (SB), U-0126 (U), SP-600125 (SP), at 15 µM, or DMSO (D) as a control. (A) Cell viability was assayed using CCK8 kit. Data are mean ± SEM. n = 4. *P<0.05, **P<0.01, ***P<0.001. (B) Effect of LMWF and MAPK inhibitors on dissipation of ΔΨm in HK2 cells. Bar diagram shows ratio of red fluorescence to green fluorescence. Data are mean ± SEM. n = 4. *P<0.05, **P<0.01, ***P<0.001. (C) Representative blottings (*up*) and relative ratios of protein levels (*down*) are shown. Each bar represents the mean ± SEM. n = 3; #*P*<0.05, ##*P*<0.01, ###*P*<0.001 vs. control, *P<0.05, **P<0.01 vs. hypoxia-reoxygenation group. (D) Representative Western blottings of mitochondrial and cytosolic cyto-c are shown.

Hypoxia for 12 h followed by reoxygenation for 3 h significantly decreased the cell viability to 46.2±1.8% (*P*<0.01) compared to control cells (Fig. 5A). LMWF (1–20 µg/ml) had a protection against hypoxia-reoxygenation induced cytotoxicity and the survival of HK2 cells was improved as the concentration of LMWF increased, with 62.2±1.6% of cells pretreated with 20 µg/ml LMWF (Fig. 5A).

Exposure of HK2 cells to hypoxia-reoxygenation resulted in dissipation of ΔΨm, which was shown as decreased ratio of ‘red’ to ‘green’ fluorescence by JC-1 staining. Cells treated with LMWF demonstrated attenuation of the dissipation of ΔΨm caused by hypoxia-reoxygenation (Fig. 5B), which indicates that LMWF could diminish hypoxia-reoxygenation induced cell apoptosis. Both p38 inhibitor, SB-203580, and JNK inhibitor, SP-600125, attenuated damage in HK2 cells exposed to hypoxia-reoxygenation as shown as the attenuation in decreased cell viability and the dissipation of ΔΨm. The ERK inhibitor, U-0126, did not have a significant effect (Fig. 5A and B).

As shown in Fig. 5C and 5D, after reoxygenation for 3 h, the pro-apoptotic proteins Bax, cleaved caspase-3 were increased, and cyto-c was released into the cytosol, whereas the anti-apoptotic protein Bcl-2 was decreased. LMWF reduced the release of cyto-c from mitochondria, and ratios of Bax/Bcl-2 and cleaved caspase-3/procaspase-3 to inhibit cell apoptosis. LMWF also inhibited p53 phosphorylation in a dose-dependent manner after reoxygenation for 3 h. SB-203580 and SP-600125 reversed up-regulation of cyto-c in the cytosol, and ratios of Bax/Bcl-2 and cleaved caspase-3/caspase-3 in HK2 cells exposed to hypoxia-reoxygenation. Further, SB-203580 also inhibited phosphorylation of p53. U-0126 did not attenuate hypoxia-reoxygenation's effect.

**Figure 6:**
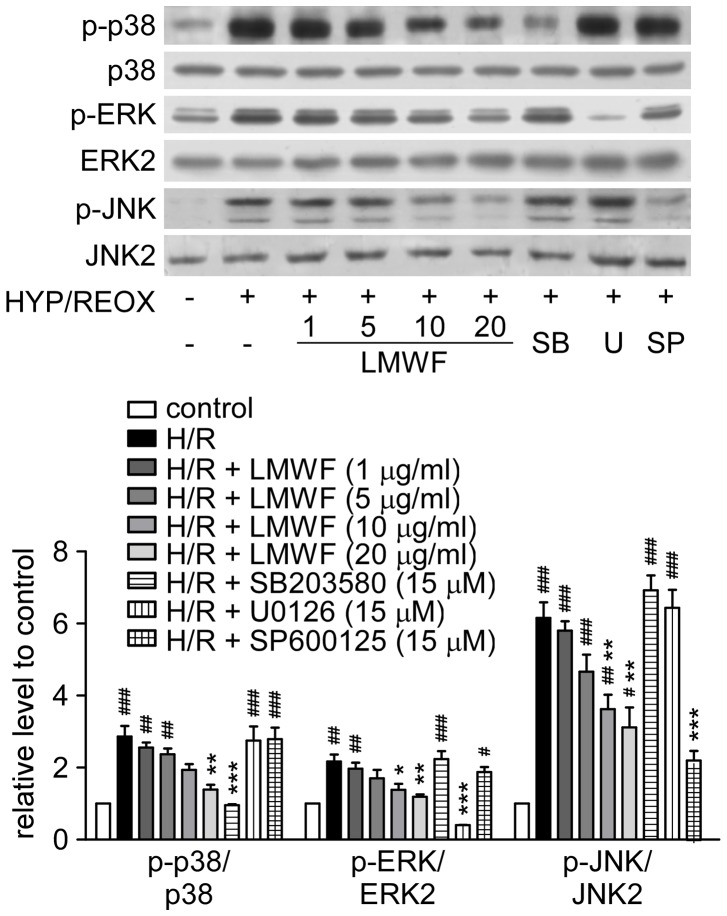
LMWF inhibited MAPK signaling pathways in HK2 cells exposed to hypoxia-reoxygenation. Expression and phosphorylation levels of ERK, p38, JNK of HK2 cells exposed to hypoxia for 12 h followed by reoxygenation for 30 min with 1∼20 µg/ml LMWF or MAPK inhibitors, SB-203580 (SB), U-0126 (U), SP-600125 (SP), at 15 µM were detected by Western blot analysis. Representative blottings (*up*) and relative ratios of protein levels (*down*) are shown. Each bar represents the mean ± SEM. n = 3; #*P*<0.05, ##*P*<0.01, ###*P*<0.001 vs. control, *P<0.05, **P<0.01, ***P<0.001 vs. hypoxia-reoxygenation group.

The phosphorylation of p38, ERK1/2, and JNK were significantly increased in HK2 cells after restoration of oxygenation for 30 min. LMWF significantly decreased the phosphorylation of p38, ERK1/2, and JNK in HK2 cells in a dose-dependent manner (Fig. 6).

### LMWF protected HK2 cell from damage caused by CoCl_2_

**Figure 7:**
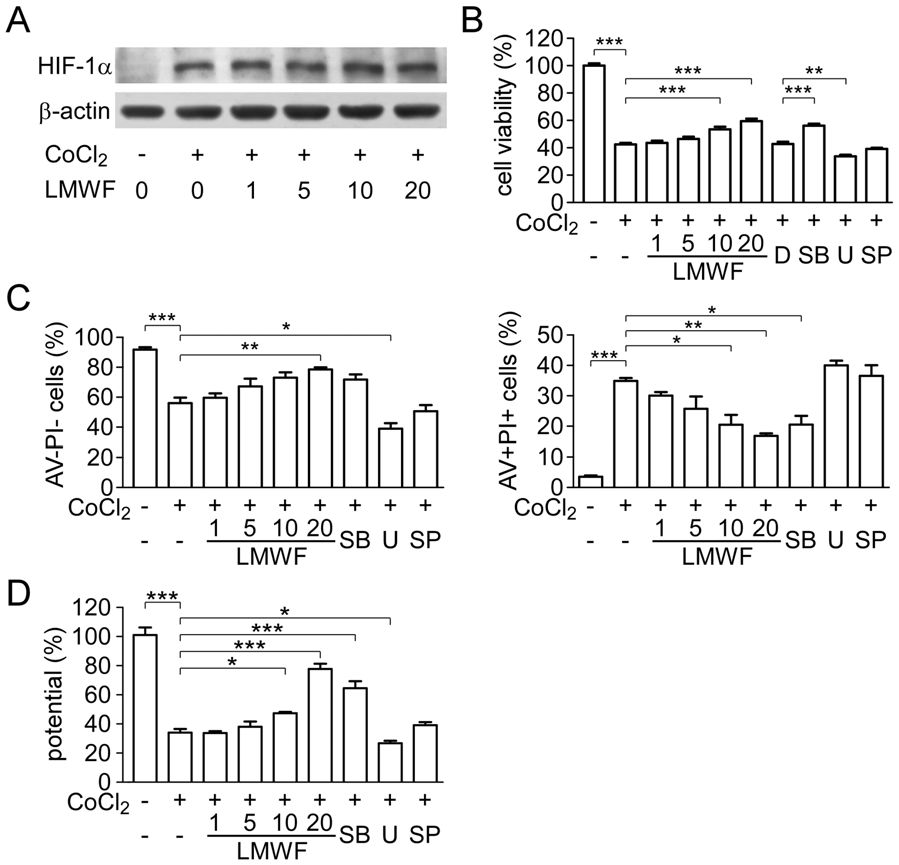
LMWF protected HK2 cells treated with CoCl_2_. Cells were cultured to 70% to 80% confluence, and then were pretreated with 1∼20 µg/ml LMWF or MAPK inhibitors SB-203580 (SB), U-0126 (U), SP-600125 (SP), at 15 µM, or DMSO (D) as a control for 1 h, and then cells were cultured in serum-free DMEM/F12 with 600 µM CoCl_2_. (A) HIF-1α protein level in HK2 cells treated with 600 µM CoCl_2_ for 3 h were detected by Western blot analysis. Representative blottings are shown. (B) Viability of HK2 cell treated with 600 µM CoCl_2_ for 24 h was assayed using CCK-8 kit. (C) Left bar diagram shows the population of live cells. Right bar diagram shows the population of late apoptotic cells. (D) Dissipation of ΔΨm in HK2 cells treated with 600 µM CoCl_2_ for 18 h. Bar diagram shows ratio of ‘red’ to ‘green’ fluorescence that represented ΔΨm of cells. Data are mean ± SEM. n = 4. *P<0.05, **P<0.01, ***P<0.001.

To confirm the protective effect of LMWF on cell damage caused by IRI, hypoxia in HK2 cells was chemically induced using CoCl_2_. As shown in Fig. 7A, the HIF-1α expression was significantly increased in cells exposed to 600 µM CoCl_2_ for 3 h. There was no difference in HIF-1α expression between LMWF-treated and untreated HK2 cells. Viability of HK2 cell treated with CoCl_2_ for 24 h was assessed using CCK-8 assay. LMWF prevented the cytotoxic effect of CoCl_2_ on HK2 cells in a dose-dependent manner (Fig. 7B).

Cell apoptosis was detected with a flow cytometer after the staining with annexin-V-FITC and PI. CoCl_2_ induced apoptosis in HK2 cells as demonstrated by the decreased number of annexin V^−^/PI^−^ cells (live cells) and the increased number of annexin V^+^/PI^+^ cells (late apoptotic cells). LMWF significantly reduced cell apoptosis in a dose-dependent manner (Fig. 7C). Exposure of HK2 cells to CoCl_2_ for 18 h resulted in dissipation of ΔΨm, which was shown as increased green fluorescence and decreased red fluorescence by JC-1 staining. As shown in Fig. 7D, HK2 cells treated with CoCl_2_ showed the lower potential, and cells pre-treated with LMWF showed attenuation of the dissipation of ΔΨm.

**Figure 8:**
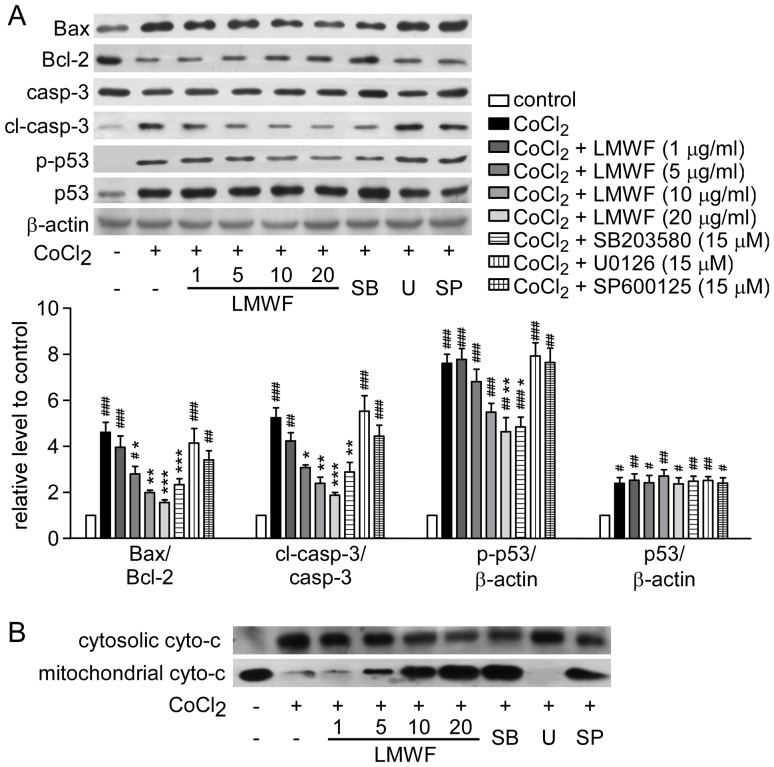
LMWF prevented apoptosis in HK2 cells treated with CoCl_2_. (A) Expression of Bax, Bcl-2, caspase 3, p-p53 and p53 of HK2 cells treated with 600 µM CoCl_2_ for 18 h were detected by Western blot analysis. Representative blotting (*up*) and quantification of protein levels (*down*) are shown. Each bar represents the mean ± SEM. n = 3; #*P*<0.05, ##*P*<0.01, ###*P*<0.001 vs. control, *P<0.05, **P<0.01, ***P<0.001 vs. CoCl_2_ treated group. (B) Representative Western blottings of mitochondrial and cytosolic cyto-c are shown.

After CoCl_2_ treatment for 18 h, the expression of pro-apoptotic proteins Bax, cleaved caspase-3 were increased, and cyto-c was released into the cytosol, whereas the expression of anti-apoptotic protein Bcl-2 was decreased (Fig. 8A, and 8B). LMWF suppressed the levels of Bax, cleaved caspase-3, and cyto-c in the cytosol, and increased the level of Bcl-2 dose-dependently. p53 was also activated in HK2 cells treated with CoCl_2_ for 18 h, LMWF inhibited phosphorylation of p53 in a dose-dependent manner.

As shown in Fig. 7, the pharmacological inhibitor of p38, SB-203580, showed attenuated damage to HK2 cells induced by CoCl_2_ in decrease level of apoptosis and attenuation the dissipation of ΔΨm, while the inhibitor of ERK, U-0126 showed reversed effect and aggravate the damage. SP-600125 did not have a significant effect on either a decrease in the cell viability or cell apoptosis induced by CoCl_2_. As shown in Fig. 8, SB-203580 reversed up-regulation of cyto-c in the cytosol, ratios of Bax/Bcl-2 and cleaved caspase-3/caspase-3 caused by CoCl_2_. Further, SB-203580 also inhibited phosphorylation of p53 in HK2 cells. These results implicated that LMWF might inhibit cell apoptosis via inhibiting p38–p53 pathway. The data showed that neither U-0126 nor SP-600125 could attenuate the effect of CoCl_2_ on cyto-c release and ratios of Bax/Bcl-2 and cleaved caspase 3/caspase 3.

**Figure 9:**
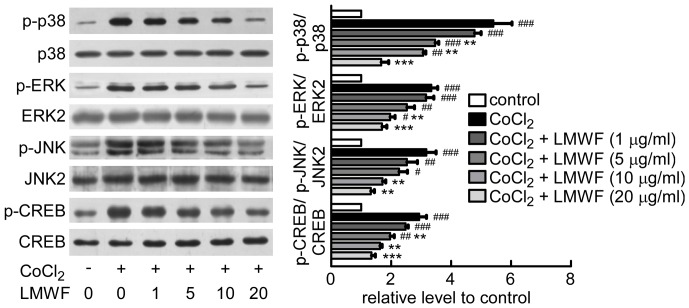
LMWF inhibited MAPK signaling pathways in HK2 cells exposed to CoCl_2_. Expression and phosphorylation levels of ERK, p38, JNK, CREB in HK2 cells treated with 600 µM CoCl_2_ for 30 min were detected by Western blot analysis. Representative blottings (*left*) and relative ratios of protein levels (*right*) are shown. Each bar represents the mean ± SEM. n = 3; #*P*<0.05, ##*P*<0.01, ###*P*<0.001 vs. control, **P<0.01, ***P<0.001 vs. CoCl_2_ treated group.

Fig. 9 shows that the phosphorylation of p38, ERK1/2, JNK, and CREB were significantly increased in HK2 cells incubated with CoCl_2_. LMWF significantly decreased phosphorylation of p38, ERK1/2, JNK, and CREB caused by CoCl_2_ in a dose-dependent manner.

## Discussion

The current study aimed to investigate the possible protective effects of LMWF against acute renal IRI. First, we evaluated the efficacy of LMWF using an *in vivo* acute renal IRI model, which is a classical model for studying the pharmacological activities of drugs on IR and related mechanisms. Murine kidneys that had undergone IR showed characteristic morphological changes, such as tubular dilatation and brush border loss, with renal dysfunction demonstrated by increased blood creatinine and BUN levels. Administration of LMWF resulted in significant improvements in renal morphology and function. LMWF also reversed the levels of MPO, MDA and SOD in renal tissue after IR.

MPO is a characteristic constituent of neutrophil granules and used widely as a biochemical marker for tissue invasion or content of neutrophils. Preventing or decreasing neutrophil invasion to postischemic tissues by blocking any step of neutrophil activation has shown to decrease tissue MPO activity. It has been reported that tissue MPO activity decreased with fucoidin treatment after rat heart IRI [18], [21]. In our study, LMWF also significantly decreased MPO activity in kidney after IRI. Moreover, treatment with fucoidin blocks infiltration of inflammatory cells [22], and inhibits leukocyte rolling and reduces leukocyte emigration in a model of sepsis [23]. Fucoidan also suppresses adipogenesis through the inhibition of major markers and inflammation-related cytokines in adipocytes [24]. These studies suggest that LMWF may protect kidney from renal damage by its anti-inflammatory effect.

Oxidative stress can result from increased ROS production, and/or from decreased ROS scavenging capability. Generation of ROS by mitochondria is a normal process as a consequence of existence in aerobic environment, but biological systems have evolved an impressive array of antioxidant enzymes like SOD, etc. to cope up with the lethal oxidative environments. SOD and other antioxidants are believed to play important roles in reversing the pathological damage caused by IRI [25]. Lipid peroxidation is implicated in the pathogenesis of postischemic tissue injuries [26]. In the study reported here, we determined oxidative injury in the kidney during IR by measuring SOD activity and MDA concentration which is the end product of lipid peroxidation and has been known as an index of tissue injury until now. MDA concentration was significantly increased in renal tissue after IR. LMWF treatment did not change the renal tissue MDA level, but significantly reduced IR-caused MDA increase. SOD activity was significantly decreased in IR renal tissue, and LMWF significantly reversed IR induced SOD changes in renal tissue, which indicates that LMWF might normalize the redox status of the renal cells under IR.

*In vitro* hypoxia-reoxygenation is known to mirror the response obtained in animal models of IR [27]. To confirm the protective effects of LMWF on IRI found in *in vivo* model, HK2 cell line was chosen to create a hypoxia-reoxygenation model and a chemical hypoxia model. HK2 cell originates from human renal tubular epithelium, which is the most susceptible to IRI. In addition, damage in the proximal epithelium is a major cause of renal dysfunction [28]. Another chemical hypoxia model induced with CoCl_2_ was also used in this study. Numerous studies have demonstrated that CoCl_2_, as a chemical hypoxia agent, can mimic the hypoxic/ischemic condition [29], [30]. In agreement with the previous studies, HK2 cells exposed to CoCl_2_ dose-dependently attenuated cell viability (data not shown). CoCl_2_ treatment causes hypoxia, a key event during IRI, to alter gene and protein expression similarly to ischemia [29], which induces hypoxia by blocking the degradation of HIF-1α and enhancing subsequent HIF-1α accumulation [31]. Moreover, CoCl_2_ also induces apoptosis. CoCl_2_ is a stimulator of the mitochondria-apoptotic pathway, which has been shown to increase intracellular ROS in diverse cell types [32], [33], [34], [35]. These studies suggest that CoCl_2_-treated HK2 cells may serve as a simple and useful *in vitro* model for exploring the mechanisms underlying the renal IRI.

Our experiments showed that LMWF had cytoprotective effect on HK2 cells. LMWF significantly reduced cell apoptosis caused by hypoxia-reoxygenation or chemical hypoxia.

Apoptosis has been shown to be an important event and a representative form of programmed cell death after renal IR [28], [36]. Apoptotic cell death is characterized by the activation of either the extrinsic pathway, which is initiated by activation of death receptors leading to activation of JNK, or the intrinsic pathway, which is marked by mitochondrial depolarization, release of cyto-c [37]. In renal cells, the mitochondrion is a key site for integrating intrinsic and extrinsic pro- and anti-apoptotic signals during stress. Increased levels of ROS have been demonstrated to induce depolarization of the mitochondrial membrane, which eventually produces an increase in the level of other pro-apoptotic molecules in cells [38]. The Bcl-2 family members are key players in the mitochondria-dependent intrinsic pathway of apoptosis. Bax is a member of the Bcl-2 family proteins, which can promote apoptosis by forming oligomers on the outer mitochondrial membrane and forming a channel for the release of cyto-c [28]. Cyto-c accumulates in the cytosol, where it participates in autoactivation of initiator caspase-9. The activated caspase-9 then proteolytically cleaves and activates executioner such as caspase-3 [39]. It has been reported that fucoidan treatment inhibited apoptosis in H_2_O_2_-induced PC12 cells by decreasing the Bax/Bcl-2 ratio and decreasing active caspase-3 expression [40]. In our study, either CoCl_2_ or hypoxia-reoxygenation induced dissipation of the ΔΨm in mitochondria, cyto-c release followed by caspase-3 activation. LMWF increased the expression of anti-apoptotic protein Bcl-2 and suppressed the expression of pro-apoptotic protein Bax both *in vivo* and *in vitro*. The Bax/Bcl-2 ratio, which helps to determine the susceptibility of cells to a death signal by regulating the function of mitochondria, was found to decrease in a dose-dependent manner by treatment with LMWF. LMWF subsequently inhibited the release of cyto-c and activation of caspase-3. Thus, LMWF affected both pro-survival and pro-apoptotic molecules with the net effect at the concentration used. The inactivation of pro-apoptosis molecules resulted in a statistically significant increase in cell viability. LMWF resulted in a significant decrease in cyto-c release, ratios of Bax/Bcl-2 and cleaved caspase-3/caspase-3, and phosphorylation of p53, which may be related to the activation of MAPK pathways.

Activation of MAPKs leads to activation of transcription factors, which are important molecular targets for pharmacological intervention and drug development because they regulate multiple genes involved in the survival or death of cells. Reperfusing the mouse kidney for 2 h, exposing HK2 cells to CoCl_2_ or reoxygenation for 30 min, caused a drastic and rapid increase in phosphorylations of JNK, p38 and ERK1/2. Pre-treatment with LMWF resulted in decreased phosphorylations of all three molecules. ERK1/2 is generally associated with cell proliferation and growth [41]. A substrate for ERK1/2 is the transcription factor CREB that, when phosphorylated at Ser133, may promote cell survival [42]. In addition to, JNK and p38 are also predominantly activated through environmental stresses, including oxidative stress, and inflammatory cytokines [41]. However, it has been reported that JNK inhibits anti-apoptotic protein Bcl-2 [43], and SP-600125 could decrease renal tubular epithelial cell apoptosis induced renal IR by inhibition of the downstream mechanism of JNK mediated apoptosis [44]. JNK and p38 could phosphorylate BH3-only proapoptotic proteins Bim and Bmf, which were thought to mediate apoptosis through a Bax dependent mitochondrial apoptotic pathway [45]. It has also been reported that apoptosis could be induced via a mitochondrial-mediated ROS–p38–p53 (ser 392 phosphorylation) pathway [46], and p38 mediated p53 phosphorylation and subsequent Bax expression in the apoptotic paradigm of cerebral endothelial cells [47]. In our study, the phosphorylation of p53 was drastically reduced in cells treated with LMWF.

Furthermore, we employed three MAPK specific inhibitors to investigate the role of MAPK pathways in cell apoptosis induced by hypoxia-reoxygenation or CoCl_2_. We found that the inhibitors of the three major MAPK pathways resulted in different results in the two cell models. SB-203580 inhibited cell apoptosis induced by either CoCl_2_ or hypoxia-reoxygenation, SP-600125 only inhibited cell apoptosis induced by hypoxia-reoxygenation, and U-0126 did not inhibit cell apoptosis, even aggravated the cell injury induced by CoCl_2_. These evidences show that LMWF may inhibit cell apoptosis via multiple mechanisms, related to an attenuation of cell death signaling pathways mainly involving p38 and JNK.

The molecular mechanisms underlying IR-induced AKI are not fully understood, but it has been suggested that the mechanisms are most likely multifactorial and interdependent, including endothelial and epithelial cell death, intratubular obstruction, changes in local microvascular blood flow, as well as immunological and inflammatory processes [28], [48], [49]. It is not excluded that LMWF may prevent kidney injury and promote kidney repair after IR by other mechanisms. Fucoidan induced mobilization of endothelial progenitor cells and their incorporation in ischemic areas for repairing the damaged tissue [50], [51]. LMWF may, therefore, be able to modulate growth factor dependent pathways in the tissue repair.

In summary, our results firstly indicated LMWF ameliorates acute renal IRI *in vivo* and *in vitro*. This protective effect led to the restoration of renal function, balance of oxidative stress and antioxidative defense, and anti-apoptosis. LMWF suppressed activation of MAPKs, which resulted in a significant decrease in cyto-c release, ratios of Bax/Bcl-2, cleaved caspase-3/caspase-3, and phosphorylation of p53 in a dose-dependent manner. The current study provides evidence that LMWF may serve as a potential therapeutic agent for acute renal IRI, and MAPK pathways especially JNK/p38-MAPK may be an appealing therapeutic target for AKI.
